# The Morphology of Impacted Maxillary Central Incisors: A Systematic Review

**DOI:** 10.3390/medicina58040462

**Published:** 2022-03-22

**Authors:** Guoda Mockutė, Gustė Klimaitė, Dalia Smailienė

**Affiliations:** 1 Faculty of Odontology, Medical Academy, Lithuanian University of Health Sciences, J. Lukšos-Daumanto 2, LT-50106 Kaunas, Lithuania; guste.klimaite@gmail.com; 2 Department of Orthodontics, Medical Academy, Lithuanian University of Health Sciences, J. Lukšos-Daumanto 6, LT-50106 Kaunas, Lithuania; dalia.smailiene@lsmuni.lt

**Keywords:** impacted, maxillary central incisors, crown length, root morphology, root length, root dilaceration

## Abstract

*Background and Objectives*: The knowledge of the morphology of impacted maxillary central incisors may lead to more effective treatment. Therefore, this systematic review aimed to evaluate the morphology of impacted maxillary central incisors and compare them with contralateral teeth. *Material and methods*: This systematic review adhered to the PRISMA statement. The literature search was carried out using PubMed (Medline database), Cochrane Library, ProQuest, Web of Science and Science Direct electronic databases with no publication date restrictions up to July 2021. Data assessing the morphology of unilaterally impacted maxillary central incisors (ICI) evaluated with CBCT were extracted, and the quality of the studies was evaluated. Crown length, root length, and root dilaceration of impacted maxillary central incisors were compared with contralateral unimpacted teeth. *Results*: The initial database search identified a total number of 287 studies. After applying the selection criteria, 21 articles were selected for a full-text analysis, and four retrospective studies involving 205 patients were included in the systematic review. According to the Newcastle-Ottawa Scale (NOS), two of included articles were graded as “Good” and the remaining two as “Fair” quality. The results showed no difference between impacted teeth and their contralateral crowns, or a minor decrease in ICI crown length (from 0.15 to 0.56 mm). The root lengths of impacted maxillary central incisors were considerably shorter than contralateral incisors (from 2.13 to 3.22 mm) and, as dental age increased, root growth decreased and the incidence of root dilaceration was more frequent. *Conclusions*: The root lengths of impacted maxillary central incisors were considerably shorter compared to the contralateral incisors. Root dilacerations frequency and severity increased as dental age increased.

## 1. Introduction

The impaction of maxillary central incisors is the third most common impaction (with an incidence of approximately 0.03–2.1%) [1,2,3] after the third molars (approx. 24.4%) [4] and upper permanent canines (approx. 2%) [5,6]. Although the impaction is relatively rare, it poses a huge challenge for both the patients and the professionals. Due to the specific location of the central incisors, their absence has a significant impact, not only on the person’s facial aesthetics, but also on function, phonetics, and psychology [7,8,9,10,11]. 

The impaction of central incisors is of multifactorial origin. The key components involved are supernumerary teeth, odontomas, and trauma. Supernumerary teeth and odontomas are the most common cause of delayed eruption of maxillary incisors with 56–60% of supernumerary teeth causing an impaction due to a direct obstruction to eruption [12,13]. Another reason for a failed eruption is tooth malformation or dilacerations. Dilacerations are often caused by trauma to a primary tooth, where the developing permanent tooth bud is affected because of the close proximity to the primary tooth. This leads to the development of root curvature in the labio-lingual or medio-distal direction [14,15]. The position of root dilaceration of the permanent central incisor depends on the developmental stage of the tooth at the time of injury [16]. In contrast, in some dilaceration cases, there are no signs of traumatic origin, therefore it is suggested that this anomaly is likely to be caused by the ectopic development of tooth buds [17]. Further possible causes of impacted maxillary central incisors may also be attributed to other morphological and positional abnormalities, such as ectopic position of the tooth germ, pathological obstructions in the eruptive path, non-vital or ankylosed primary incisors, early loss of deciduous teeth, mucosal barriers, endocrine abnormalities, and bone disease [18,19,20,21].

Diagnosis of the impacted or nonerupted teeth is usually made based on clinical and radiographic findings. Retention of the primary tooth, insufficient space in the region of an unerupted tooth, late eruption, and atypical elevation of the soft tissue of the palatal or labial mucosa are all clinical symptoms of an impacted tooth [22]. Radiologic evaluation is necessary to confirm the presence of tooth impaction, the position and orientation of the impacted tooth, and the possibility of the adjacent teeth root resorption. However, preoperative determination of the morphology of an unerupted tooth is also an important factor in diagnosis and treatment planning. According to Lin et al. [23], the prognosis for orthodontic traction is better for a tooth in a lower position in relation to the alveolar crest, dilacerated root with an obtuse inclination angle, and incomplete root development. Conventional radiography cannot always demonstrate structures in all three planes, and frequently an image is obscured because of superimposition on other structures. Bodner et al. [24] analysed image accuracy of conventional radiography and computerized tomography (CT) when assessing impacted teeth. The result showed that the crown shape, root shape, crown/root relationship, and tooth inclination were significantly more clearly shown on CT than they were on two-dimensional radiography. Lately, cone-beam computed tomography (CBCT) is widely used as a diagnostic tool for impacted teeth. Not only are the effective doses smaller than those of medical CT, but also it is more time-efficient, more cost-effective, and still able to provide 3D images with an unlimited number of views [25,26].

This systematic review aims to summarize all currently available data pertaining to the morphology of impacted maxillary central incisors evaluated by CBCT.

## 2. Materials and Methods

This systematic review was conducted and reported according to the PRISMA statement [27]. This review’s protocol was set a priori and registered in PROSPERO (registration number: CRD42021252978).

### 2.1. Search Strategy and Study Selection Criteria

The literature search was conducted using five electronic databases: PubMed (Medline database), Cochrane Library, ProQuest, Web of Science and Science Direct. In order to ensure all relevant material were captured, the reference lists of included studies or relevant reviews identified through the search were scanned. According to the Participants Intervention Comparison Outcome Study design (PICOS) schema [28], the review aimed to investigate human patients of any age, sex, ethnicity, or malocclusion with unilateral impaction of maxillary central incisors. The analysis of radiological evaluation (CBCT) measurements was performed to compare the impacted central incisor with unimpacted contralateral central incisor (CCI). The primary outcome evaluated was morphological characteristics of untreated impacted maxillary central incisors: crown length, root length and root dilaceration (angle, position). Medical Subject Headings (MeSH) terms used were “impacted maxillary central incisors” combined with “crown length”, ”root length”, ”root morphology”, and ”root dilaceration”. The last search was executed in August 2021.

### 2.2. Eligibility Criteria

Eligibility criteria are listed in Table 1. There were no restrictions set regarding the year of publication, gender, age, or population.

### 2.3. Selection Process and Data Collection Process

**Figure 1:**
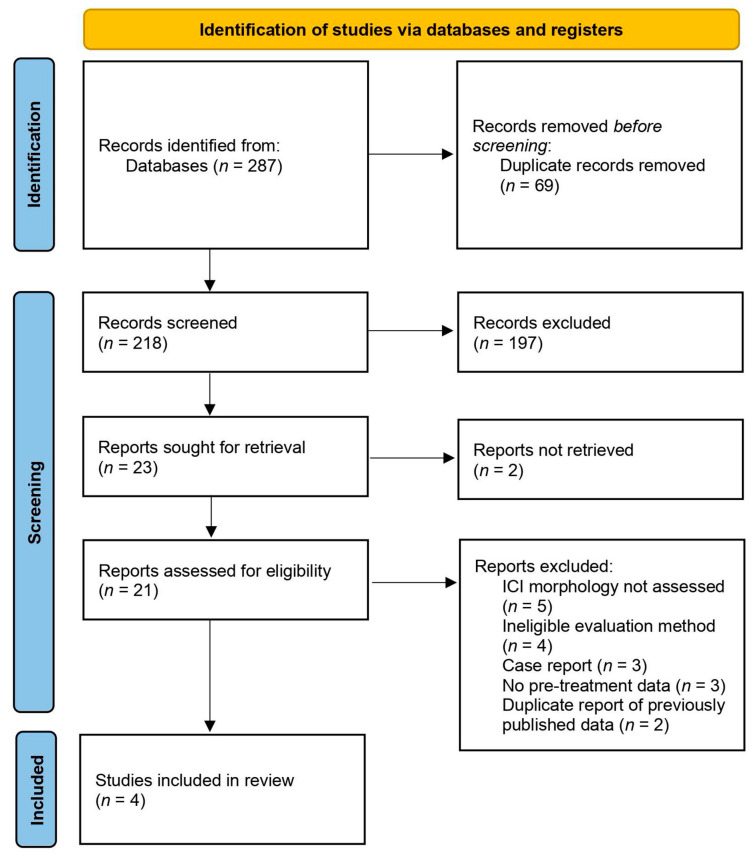
PRISMA flow diagram for the identification and selection of eligible studies.

The literature search was performed by two peers independently and reviewed by a third. Primary articles were screened according to title and abstract. The preselected studies that included relevant information were downloaded as full texts and evaluated according to eligibility criteria. The results of the search were discussed, any disagreements were resolved by discussion and mutual consensus or by consultation with a third reviewer. The PRISMA flow diagram provides detailed information regarding the selection process of studies (Figure 1).

### 2.4. Data Extraction

Data from studies that met the inclusion and exclusion criteria were extracted independently by each reviewer. Data extracted from each article included the following: (1) author, year of study; (2) study design; (3) number, age, gender of patients; (4) the comparison group; (5) eligible outcome and morphological characteristics (crown lengths, root lengths and root dilacerations (incidence, angle, position)).

### 2.5. Quality Assessment

The Newcastle-Ottawa Scale (NOS) risk of bias assessment tool for observational studies recommended by the Cochrane Collaboration to assess quality of included studies [29]. The NOS allocates up to a maximum of nine points (stars) for the least risk of bias in three domains: (1) selection of study groups (four points); (2) comparability of groups (two points); and (3) ascertainment of outcomes (three points). Stars awarded for each quality item serve as a quick visual assessment.

## 3. Results

### 3.1. Study Selection

The electronic database search identified 287 records of our interest. After duplicate removal, 218 records remained, which were screened for relevance. Screening of titles and abstracts led to the exclusion of 197 studies that did not meet the inclusion criteria. Additionally, it was not possible to retrieve two full-text reports that were unavailable in English. Of the 21 full-text articles we assessed for eligibility, a further 17 studies were excluded (reasons of exclusion are listed in Figure 1). Finally, four studies were included in a systematic review. An overview of the search results and screening process is summarized in the study flow chart (Figure 1).

### 3.2. Study Characteristics

All articles included were retrospective studies with a split-mouth design. Fundamental data extracted from individual studies are presented in Table 2. The average number of patients per study was approximately 51 patients (with a minimum of 26 and a maximum of 108 patients). The total number of included children was 205 (103 females and 102 males), with an age range from 6.5 to 16 years old. Dilacerated ICIs were included in all studies, however, Rizzatto et al.’s [30] study did not include severely dilacerated teeth. The pre-treatment CBCT images were analysed to compare impacted maxillary central incisors with unimpacted CCI. Three studies evaluated root lengths (mm) using the analogous methodology with the same landmarks (cementoenamel junction (CEJ) and the root apex) [30,31,32]. If the dilacerations were prominent, the root lengths were measured in three straight segments, following the root canal [30], in a line following the curvature [31], or separated into two parts (dilacerated and non-dilacerated) [32]. Sun et al. [32] and Lyu et al. [33] reported data of root dilaceration, including angle, position, and frequency. Rizzatto et al. [30], Sun et al. [32], and Lyu et al. [33] indicated crown lengths (mm), measured from incisal edge to CEJ. The position of impacted central incisors was evaluated by measuring the inverse angle (angle between the long axis of the crown and palatal plane) [32], or by the direction of eruption (palatal, labial, nasal) [33].

Three studies [30,32,33] received ethical approval from their ethical committee/review board, yet no information regarding this matter was mentioned by Shi et al. [31]. Informed consent was obtained in all included studies.

### 3.3. Results of Individual Studies

The outcomes from included studies are presented in Table 3. Three studies evaluated the crown lengths of impacted central incisors [30,32,33]. Rizzatto et al. [30] reported that crown lengths of ICI were significantly shorter than in contralateral incisors by 0.56 mm (*p* < 0.001). Similarly, Sun et al. [32] found that the crown lengths of ICI were statistically shorter (by 0.21 mm and 0.15 mm, respectively) in both early and late dental age groups compared to CCI. Lyu et al. [33] analysed the crown lengths of dilacerated and non-dilacerated central incisors groups, however, a significant difference compared to contralateral incisors was not observed.

Three studies included in the present systematic review reported measurements of root lengths [30,31,32]. Rizzatto et al. [30] and Shi et al. [31] found that the pre-treatment root lengths of ICI were significantly shorter compared to CCI by 3.22 mm and 2.35 mm, respectively (*p* < 0.001). Sun et al. [32] reported that both early and late dental age groups indicated that the root lengths of ICI were considerably shorter than those of CCI by 2.13 mm and 2.79 mm, respectively (*p* < 0.05). The measurements of root length in Lyu et al. [33] article were not specified. To retrieve the information regarding reliable root lengths, the authors were contacted by e-mail, however, no response was received.

Root morphology in terms of dilaceration was evaluated in two studies conducted by Sun et al. [32] and Lyu et al. [33]. Sun et al. [32] discovered that the incidence of dilaceration increases with patient age. The early dental age group showed 50% of dilacerations while the late dental age group—about twice that more 95.5% (*p* < 0.001). Lower incidence of dilaceration was established in a study by Lyu et al. [33]. Dilacerations were the most common in labially impacted teeth (42.6%). Regarding the localization of dilaceration, the results of Lyu et al. [33] show that the dilacerations in the middle third of the root were the most common. In contrast, Sun et al. [32] noted the highest occurrence of dilacerations in the CEJ (25%) and cervical third of root (32.5%) areas.

While evaluating the location of root dilaceration, Lyu et al. [33] established that it was statistically different among nasal, labial, and palatal impaction groups (*p* < 0.01). The majority of labially ICI had root dilacerations in the middle third (23.1%) and apical third (13.0%) of the root. The palatally ICI dilacerated roots had severely curved angles at the cervical thirds (78.3%). Nasally impacted teeth were indicated with an acute curvature of the apical third of the root (43.8%) [33]. Lyu et al. [33] presented an additional measurement of dilaceration position confirming that impacted incisors with a nasal impaction had a statistically higher dilaceration position (9.80 mm (1.29)) compared to others. Results were also confirmed by measurement of K-value (the ratio between the available length of the direct root and the ideal length of the direct root). According to investigators, the lower the K-value, the nearer the dilacerated position is to the cervix which may have a worse prognosis for orthodontic traction [33]. The highest K-value was noticed in nasally impacted teeth with non-dilacerated roots (1.38–1.52), the lowest K-value was indicated in palatally impacted incisors with dilacerated roots (0.16–0.19).

The size of dilaceration angle (angle between the two dilacerated parts of the root) was observed to be significantly larger in the early dental age group compared to the late dental age group by 32.75° [32]. Lyu et al. [33] assessed that the size of root dilaceration angle was statistically different among nasal, labial, palatal impaction groups (*p* < 0.01). In labially impacted teeth the highest dilaceration angle (112.46° (9.67)) was recorded.

In multiple linear regression analyses, Sun et al. [32] estimated that the position of impacted tooth (evaluated by measuring the inverse angle) did not correlate with the root length. However, the inverse angle in the late dental age group was greater by 6.5° on average compared to the early dental age group.

### 3.4. Quality Assessment

Table 4 outlines the risk of bias evaluations as scored by the Newcastle-Ottawa scale. Ratings of our included studies ranged from 5 to 7 out of a possible 9 points. Two selected articles were rated as “Good” quality and low risk of bias [31,32], whereas the remaining two ranked as “Fair” quality and moderate risk of bias [30,33]. Most points were not obtained due to a lack of controls for possible confounders as well as ascertainment of exposure.

## 4. Discussion

Evaluating root morphology is imperative for rational treatment planning, particularly selecting appropriate therapeutic timing and protocol of impacted maxillary central incisors, as well as the probability of a spontaneous eruption. Treatment of upper permanent impacted incisors includes early interceptive measures to facilitate the eruption of displaced maxillary incisors or surgical exposure of the tooth’s crown with the subsequent orthodontic alignment of the tooth. A successful alignment of an impacted tooth depends on several factors: the position and direction of the ICI, the degree of root formation, the degree of dilacerations, and the presence of space available for the impacted tooth [23,34,35,36,37,38]. In various studies, the recorded rate of spontaneous eruption ranges from 30.3% to 89.4% of cases and is dependent on the initial maturation of the impacted tooth’s root, initial vertical position, and degree of angulation of the impacted incisor, the form of the obstacle, and additional orthodontic expansion of the dental arch [39,40,41,42]. However, in some cases, impacted incisors do not erupt, and surgical-orthodontic treatment is needed. In a recent systematic review and meta-analysis evaluating spontaneous eruption of impacted maxillary incisors, a clinical recommendation was made to wait for the eruption of the tooth for a period of 12–36 months after surgical removal of the obstacle impeding the eruption of permanent tooth [43]. The calculated average eruption potential of impacted anterior maxillary teeth following such procedure was approximately 65.5%, with a higher odds ratio for patients under nine years of age. However, the present analysis shows that the root of the impacted incisor could achieve better development if treated early. The selected articles showed that root lengths of impacted maxillary central incisors are considerably shorter compared to contralateral incisors by 2.13 to 3.22 mm [30,31,32]. The results also show a trend that with age the development of the root increasingly lags behind. Sun et al. [32] analysed two dental age groups: the early dental age group included teeth with a third or two-thirds of its root formation, and the late dental age group was defined as teeth with an almost complete root with an open apex or completion of the root’s apical end. Though it was stated that roots continued to grow as dental age increased, the difference between root lengths of impacted and normally erupted incisors was larger in the late dental age group. The same is observed when comparing Rizzatto et al.’s [30] and Shi et al.’s [31] results. The Rizzatto et al. [30] study included older patients (mean age 9.5 years, range 7–14) than Shi et al.’s [31] study (mean age 8.44 years, range 6.5–11.2), therefore, the difference between root lengths of impacted and normally erupted incisors was larger in the Rizzatto et al. [30] study (3.22 mm and 2.35 mm, respectively). As the measurement methodologies in the studies were analogous, it could be concluded that root formation slows down if the impacted tooth is left untreated. Root lengths also were positively associated with the length of the non-dilacerated portion of the root [32].

Not only root growth decreases, but also incidence and severity of root dilaceration increase as dental age increases because the root develops in an irregular direction. A significantly higher dilaceration angle was observed in the early dental age group than in the late dental age group by 32.75° [32]. The more the angle is obtuse, the less the root is distorted. It is possible to speculate that the earlier the dilaceration begins to form, the closer it is located to the cervix, therefore limited space is available for further root development. However, results about the localization of dilaceration are conflicting [32,33]. This may be due to different study samples. Moreover, none of the analysed studies included the dilaceration measurements of contralateral incisors, due to that the comparison was not possible.

The aetiology of dilacerations is yet not fully explained, however, there are two possible versions: trauma or idiopathic developmental disturbances [44]. It is claimed that root dilaceration is usually prominent in affected permanent maxillary incisors due to its close topographic relationship with deciduous teeth, which are commonly injured [45]. The injured Hertwig’s epithelial root sheath produces dentin at the same rate as before the injury and tends to grow in an atypical upward and lingual direction independently of its crown direction [44]. Studies point out that early management of ICI is needed because it is easier to treat the inverse tooth with shorter roots as the centre of rotation is nearer to the cervix of the tooth [32]. After early correction, the impacted tooth’s root can grow in a proper way [46].

Lyu et al. [33] stated the importance of exploring the different types of impactions to determine effective prevention and treatment for dilacerated ICI. The treatment and prognosis of teeth differ with the direction of the crown, degree of dilacerations, root formation stage, and position [23,33]. The nearer the dilacerated position is to the cervix, the worse the prognosis for orthodontic traction. In contrast, obtuse angle root dilacerations and incomplete root formation are prone to success [23]. Both Sun et al. [32] and Rizzatto et al. [30] strongly recommended starting the treatment as soon as possible for the purpose of eliminating the aetiological factors, making room for the impacted tooth’s full root development, and facilitating future treatment. Lyu et al. [33] suggested that treatment should begin no later than the closure of the apical foramen in impacted ones. Adequate treatment time allows Hertwig’s epithelial root sheath to be redirected, allowing the root to develop normally [44]. Additionally, early treatment corrects an inverse tooth more easily since the root is shorter [32]. Late treatment can result in delayed tooth eruption, midline shift, migration of adjacent teeth, loss of alveolar bone crest, and other obstacles for future treatment [47].

Regarding crown length, results of studies show no difference between impacted teeth and their contralateral crowns [33], or a minor decrease in ICI crown length (from 0.15 to 0.56 mm) [30,32], which although statistically significant, is not that clinically important.

Only one of the included articles disclosed the limitations of their study [33]. All articles were retrospective cohort studies, and the quality was graded as either “Good” or “Fair”. The main limitations of the included articles were nonhomogeneous study design and small sample sizes, as well as blinding of assessors. None of the articles calculated a reliable sample size. Due to the low prevalence of the anomaly, it is difficult to collect study groups of sufficient size. Another limitation of the analysis was that the patient population was not the same across studies. Lyu et al. [33] reported that as the anatomy of teeth varies among different populations, the measurements may not apply to other ethnic groups in different regions. This statement can be attributed to all the studies included in our research. Overall, a huge lack of clinical studies regarding the subject of root morphology of ICI was observed. This systematic review has clinical application as it provides information regarding morphological modifications of the root and crown, different types of maxillary central incisor impactions, and the impact of patient age, all of which are crucial for rational treatment planning.

## 5. Conclusions

Studies demonstrated an inconsequential difference between the crown lengths of impacted maxillary incisors and contralateral incisors. Contrarily, the impacted maxillary central incisors’ root lengths were significantly shorter compared to the contralateral incisors. Moreover, the incidence of root dilaceration increased as dental age increased. However, results concerning the localization of dilaceration are conflicting, further research with larger populations is needed for more reliable conclusions and clinical guidelines.

## Figures and Tables

**Table 1:** Eligibility criteria.

Inclusion Criteria	Exclusion Criteria
Randomized, prospective, and retrospective studies published in English	Literature reviews, case reports and series
Patients diagnosed with unilateral impaction of maxillary central incisors	Bilateral impaction of maxillary central incisors
CBCT images with radiological evaluation measurements of ICI before treatment	Panoramic or dental radiographs used for ICI evaluation
Comparison between pre-treatment measurements of crown lengths, root lengths, root dilacerations (angle, position) of ICI and naturally erupted CCI	Studies on patients with genetic syndromes (craniofacial syndromes, cleft lip, or palate), severe facial malformations or systemic diseases

**Table 2:** Study characteristics.

Authors	Study Design	Study Sample: Patients (M/F); Mean Age (SD) at T0	Comparison	Eligible Outcome
**Rizzatto et al., 2017 [30]**	RS	26 (15/11) mean age 9.5 (SD not defined) years (range 7–14) ICI with angle of crown-root dilaceration beyond 60 degrees were excluded	ICI and CCI (T0)	- Root length (dilacerated roots lengths were measured in three straight segments, following the root canal) (mm) - Crown length (mm)
**Shi et al., 2015 [31]**	RS	30 (20/10) mean age 8.44 (1.20) years (range 6.5–11.2)	ICI and CCI (T0)	- Root length (dilacerated roots lengths were measured in a line following the curvature) (mm)
**Sun et al., 2014 [32]**	RS	41 (19/22) mean age 8.69 (1.36) years (range not defined) Labial inversely ICI ICI groups: Early dental age group (*n* = 18); dental age 7.50 (0.51) Late dental age group (*n* = 22); dental age 9.55 (0.51)	ICI and CCI (T0)	- Root length (dilacerated roots lengths measurements included two parts) (mm) - Crown length (mm) - Inverse angle (angle between the long the axis of the crown and palatal plane) - Dilaceration angle (angle between the two dilacerated parts of the root) (°) - Length of non-dilaceration part of root - Dilaceration position - Dilaceration frequency
**Lyu et al., 2018 [33]**	RS	108 (60/48) mean age 11.8 (2.6) years (range 8–16) ICI groups: Palatal impaction (*n* = 23); Labial impaction (*n* = 69); Nasal impaction (*n* = 16); Dilacerated 71 (65.7%); Non-dilacerated 37 (34.3%).	ICI and CCI (T0)	- Crown length (mm) - K-value: the available length of the direct root (aLR)/the ideal length of the direct root (iLR) in the long axis of the crown - Direction of eruption (palatal, labial, nasal) - Dilaceration angle (the angle between the longitudinal axis of the crown and that of the dilacerated portion of the root) (°) - Dilaceration frequency

RS, Retrospective study; ICI, impacted central incisor; CCI, contralateral central incisor; T0, Before treatment.

**Table 3:** Outcomes.

Authors	Crown Length (mm), mean (SD)	Root Length (mm), mean (SD)	Dilaceration (Incidence, Position (mm), Angle (°))	Conclusions
**Rizzatto et al., 2017 [30]**	ICI 10.02 (1.31) CCI 10.58 (1.08)	ICI 9.21 (1.70) CCI 12.42 (1.53)	Not evaluated	Crown and root lengths were statistically shorter (0.56 mm and 3.22 mm, respectively) in ICI when compared to CCI (*p* < 0.001). Crown length of ICI was shorter in 80 % of the sample. Root length of ICI was shorter in 96 % of the sample.
**Shi et al., 2015 [31]**	Not evaluated	ICI 6.67 (1.94) CCI 9.02 (2.13)	Not evaluated	The mean pretreatment root length of ICI was statistically shorter by 2.35 mm than root length of CCI (*p* < 0.001).
**Sun et al., 2014 [32]**	ICI (early dental age group) 11.08 (1.02) CCI (early dental age group) 11.29 (0.86) ICI (late dental age group) 10.90 (0.66) CCI (late dental age group) 11.05 (0.95)	ICI (early dental age group) 5.47 (1.35) CCI (early dental age group) 7.60 (1.53) ICI (late dental age group) 7.63 (2.00) CCI (late dental age group) 10.42 (1.95)	**Dilaceration frequency:** early dental age group 50% late dental age group 95.5% **Dilaceration position:** CEJ 25% Cervical third of root 32.5% Middle third of root 12.5% Apical third of root 5% **Dilaceration angle:** early dental age group 142.43° (39.25) late dental age group 109.68° (26.03)	The crown lengths of ICI in early and late dental age groups were significantly shorter compared to CCI by 0.21 mm and 0.15 mm, respectively. The root lengths of ICI were considerably shorter than those of CCI in both the early and late dental age groups by 2.13 mm and 2.79 mm, respectively (*p* < 0.05). Incidence of dilacerations was higher in the late dental age group compared with the early dental age group (*p* < 0.001). The cervical third of the root indicated a statistically higher occurrence of dilacerations than other portions of the root. A significantly higher dilaceration angle was observed in early dental age group than in late dental age group by 32.75°.
**Lyu et al., 2018 [33]**	ICI (dilacerated group) 10.48 (0.38) ICI (non-dilacerated group) 10.49 (0.42) CCI 10.51 (0.23)	Not evaluated	**Dilaceration frequency:** Palatal impaction: 16.7% Labial impaction: 42.6% Nasal impaction: 6.4% **Dilaceration position D (mm):** Palatal impaction: 2.13 (0.32) Labial impaction: 4.33 (0.45) Nasal impaction: 9.80 (1.29) **Dilaceration angle:** Palatal impaction: 88.47° (6.28) Labial impaction: 112.46° (9.67) Nasal impaction: 59.83° (7.27)	There was no notable difference between crown lengths of dilacerated, non-dilacerated ICI and CCI. 65.7% of ICI had dilacerations greater than 20°. A significant reduction in root growth was observed in both the dilacerated and non-dilacerated ICI compared with CCI (*p* < 0.001). The dilacerations were the most incidence in labially impacted incisors.

ICI, impacted central incisor; CCI, contralateral central incisor.

**Table 4:** Quality assessment of the included studies according to Newcastle-Ottawa Scale (NOS).

Authors (Year)	Selection	Comparability	Outcome	Total Stars
Lyu et al. (2018) [33]	★★★	★	★	5
Rizzatto et al. (2017) [30]	★★★	★	★★	6
Shi et al. (2015) [31]	★★★★	★	★★	7
Sun et al. (2014) [32]	★★★★	★	★★	7

Stars (★) are assigned to each item if the requirement is satisfied.

## Data Availability

Not applicable.

## References

[1] Grover P.S., Lorton L. (1985). The incidence of unerupted permanent teeth and related clinical cases. Oral Surg. Oral Med. Oral Pathol..

[2] Gisakis I.G., Palamidakis F.D., Farmakis E.R., Kamberos G., Kamberos S. (2011). Prevalence of impacted teeth in a Greek population. J. Investig. Clin. Dent..

[3] Al-Zoubi H., Alharbi A.A., Ferguson D.J., Zafar M.S. (2017). Frequency of impacted teeth and categorization of impacted canines: A retrospective radiographic study using orthopantomograms. Eur. J. Dent..

[4] Carter K., Worthington S. (2016). Predictors of Third Molar Impaction: A Systematic Review and Meta-analysis. J. Dent. Res..

[5] Thilander B., Jakobsson S.O. (1968). Local factors in impaction of maxillary canines. Acta Odontol. Scand..

[6] Ericson S., Kurol J. (1986). Longitudinal study and analysis of clinical supervision of maxillary canine eruption. Community Dent. Oral Epidemiol..

[7] Snow K. (1961). Articulation Proficiency in Relation to Certain Dental Abnormalities. J. Speech Lang Hear. Disord..

[8] Riekman G.A., el Badrawy H.E. (1985). Effect of premature loss of primary maxillary incisors on speech. Pediatr. Dent..

[9] Bankson N.W., Byrne M.C. (1962). The Relationship Between Missing Teeth and Selected Consonant Sounds. J. Speech Lang Hear. Disord..

[10] Shaw W.C., Rees G., Dawe M., Charles C.R. (1985). The influence of dentofacial appearance on the social attractiveness of young adults. Am. J. Orthod..

[11] Rodd H., Barker C., Baker S., Marshman Z., Robinson P. (2010). Social judgements made by children in relation to visible dental trauma. Dent. Traumatol..

[12] Mathias M.F., Lobo-Piller R.G., Corrêa M.S. (2011). Treatment of supernumerary teeth. Eur. J. Paediatr. Dent..

[13] Gregg T.A., Kinirons M.J. (1991). The effect of the position and orientation of unerupted premaxillary supernumerary teeth on eruption and displacement of permanent incisors. Int. J. Paediatr. Dent..

[14] Huber K., Suri L., Taneja P. (2008). Eruption Disturbances of the Maxillary Incisors: A Literature Review. J. Clin. Pediatr. Dent..

[15] Zilberman Y., Fuks A., Ben Bassat Y., Brin I., Lustmann J. (1986). Effect of trauma to primary incisors on root development of their permanent successors. Pediatr. Dent..

[16] Ben Bassat Y., Fuks A., Brin I., Zilberman Y. (1985). Effect of trauma to the primary incisors on permanent successors in different developmental stages. Pediatr. Dent..

[17] McNamara T., Woolfe S.N., McNamara C.M. (1998). Orthodontic management of a dilacerated maxillary central incisor with an unusual sequela. J. Clin. Orthod..

[18] Vastardis H. (2000). The genetics of human tooth agenesis: New discoveries for understanding dental anomalies. Am. J. Orthod. Dentofac. Orthop..

[19] Jones J.W. (1999). A medico-legal review of some current UK guidelines in orthodontics: A personal view. Br. J. Orthod..

[20] Bodenham R.S. (1967). The treatment and prognosis of unerupted maxillary incisors associated with the presence of supernumerary teeth. Br. Dent. J..

[21] Jones J.W., Husain J. (1996). Management of the unerupted incisor. Dent. Update.

[22] Duncan W.K., Ashrafi M.H., Meister F., Pruhs R.J. (1983). Management of the nonerupted maxillary anterior tooth. J. Am. Dent. Assoc..

[23] Lin Y.J. (1999). Treatment of an impacted dilacerated maxillary central incisor. Am. J. Orthod. Dentofac. Orthop..

[24] Bodner L., Bar-Ziv J., Becker A. (2001). Image accuracy of plain film radiography and computerized tomography in assessing morphological abnormality of impacted teeth. Am. J. Orthod. Dentofac. Orthop..

[25] Abdelkarim A. (2019). Cone-Beam Computed Tomography in Orthodontics. Dent. J..

[26] Chaushu S., Chaushu G., Becker A. (2004). The role of digital volume tomography in the imaging of impacted teeth. World J. Orthod..

[27] Page M.J., McKenzie J.E., Bossuyt P.M., Boutron I., Hoffmann T.C., Mulrow C.D., Shamseer L., Tetzlaff J.M., Akl E.A., Brennan S.E. (2021). The PRISMA 2020 statement: An updated guideline for reporting systematic reviews. Int. J. Surg..

[28] Amir-Behghadami M., Janati A. (2020). Population, Intervention, Comparison, Outcomes and Study (PICOS) design as a framework to formulate eligibility criteria in systematic reviews. Emerg. Med. J..

[29] Wells G., Shea B., O’Connell D., Peterson J., Welch V., Losos M., Tugwell P. (2000). The Newcastle-Ottawa Scale (NOS) for Assessing the Quality of Non-Randomized Studies in Meta-Analysis. http://www.ohri.ca/programs/clinical_epidemiology/oxford.asp.

[30] Rizzatto S., de Menezes L.M., Rabin P., Petersen R.C., Mattiello F.D., de Lima E.M. (2017). Crown and Root Lengths of Impacted Maxillary Central Incisors and Contralateral Teeth Evaluated with Cone Beam Computed Tomography. Pesqui. Bras. Odontopediatr. Clin. Integr..

[31] Shi X., Xie X., Quan J., Wang X., Sun X., Zhang C., Zheng S. (2015). Evaluation of root and alveolar bone development of unilateral osseous impacted immature maxillary central incisors after the closed-eruption technique. Am. J. Orthod. Dentofac. Orthop..

[32] Sun H., Wang Y., Sun C., Ye Q., Dai W., Wang X., Xu Q., Pan S., Hu R. (2014). Root morphology and development of labial inversely impacted maxillary central incisors in the mixed dentition: A retrospective cone-beam computed tomography study. Am. J. Orthod. Dentofac. Orthop..

[33] Lyu J., Lin Y., Lin H., Zhu P., Xu Y. (2018). New clues for early management of maxillary impacted central incisors based on 3-dimensional reconstructed models. Am. J. Orthod. Dentofac. Orthop..

[34] Brin I. (1982). The unerupted maxillary central incisor: Review of its etiology and treatment. J. Dent. Child..

[35] Wasserstein A., Tzur B., Brezniak N. (1997). Incomplete canine transposition and maxillary central incisor impaction—A case report. Am. J. Orthod. Dentofac. Orthop..

[36] Tanaka E., Watanabe M., Nagaoka K., Yamaguchi K., Tanne K. (2001). Orthodontic traction of an impacted maxillary central incisor. J. Clin. Orthod..

[37] Kolokithas G., Karakasis D. (1979). Orthodontic movement of dilacerated maxillary central incisor: Report of a case. Am. J. Orthod..

[38] Uematsu S., Uematsu T., Furusawa K., Deguchi T., Kurihara S. (2004). Orthodontic treatment of an impacted dilacerated maxillary central incisor combined with surgical exposure and apicoectomy. Angle Orthod..

[39] Pavoni C., Franchi L., Laganà G., Baccetti T., Cozza P. (2013). Management of impacted incisors following surgery to remove obstacles to eruption: A prospective clinical trial. Pediatr. Dent..

[40] Bryan R.A., Cole B., Welbury R. (2005). Retrospective analysis of factors influencing the eruption of delayed permanent incisors after supernumerary tooth removal. Eur. J. Paediatr. Dent..

[41] Mason C., Azam N., Holt R.D., Rule D.C. (2000). A retrospective study of unerupted maxillary incisors associated with supernumerary teeth. Br. J. Oral Maxillofac. Surg..

[42] Foley J. (2004). Surgical removal of supernumerary teeth and the fate of incisor eruption. Eur. J. Paediatr. Dent..

[43] Pescia R., Kiliaridis S., Antonarakis G.S. (2020). Spontaneous eruption of impacted maxillary incisors after surgical extraction of supernumerary teeth: A systematic review and meta-analysis. Clin. Oral Investig..

[44] Topouzelis N., Tsaousoglou P., Pisoka V., Zouloumis L. (2010). Dilaceration of maxillary central incisor: A literature review. Dent. Traumatol..

[45] Asokan S., Rayen R., Muthu M.S., Sivakumar N. (2004). Crown dilaceration of maxillary right permanent central Incisor—A case report. J. Indian Soc. Pedod. Prev. Dent..

[46] Sun H., Hu R., Ren M., Lin Y., Wang X., Sun C., Wang Y. (2016). The treatment timing of labial inversely impacted maxillary central incisors: A prospective study. Angle Orthod..

[47] Tsai T. (2002). Surgical repositioning of an impacted dilacerated incisor in mixed dentition. J. Am. Dent. Assoc..

